# Efficacy and safety of taxane-based systemic chemotherapy of advanced gastric cancer: A systematic review and meta-analysis

**DOI:** 10.1038/s41598-017-05464-0

**Published:** 2017-07-13

**Authors:** Jinxin Shi, Peng Gao, Yongxi Song, Xiaowan Chen, Yuan Li, Changwang Zhang, Hongchi Wang, Zhenning Wang

**Affiliations:** Department of Surgical Oncology and General Surgery, First Hospital of China Medical University, 155 North Nanjing Street, Heping District, Shenyang, 110001 China

## Abstract

Taxanes are chemotherapeutic agents commonly used to treat several cancers. However, the effects of taxanes on advanced gastric cancer (AGC) are still not clear, especially when used as a first-line treatment. This systematic review and meta-analysis aims to investigate the efficacy and safety of taxanes as a first-line treatment of AGC. The quality of our included studies was assessed using the Cochrane risk of bias tool for RCTs and NOS scale for nRCTs, and the data of the included studies was of satisfactory quality to analyze. The outcomes included overall survival (OS), progression-free survival (PFS), overall response rate (ORR), and toxicity. Taxanes significantly improved OS (HR = 0.84, 95% CI 0.76–0.92, P = 0.0004) and had a slight effect on ORR (RR = 1.23, 95% CI 1.00–1.51, P = 0.05). However, taxanes may also increase the risks of neutropenia and leucopenia, similar to effects observed in other conventional chemotherapeutic treatments such as oxaliplatin and epirubicin. Therefore, patient characteristics including concomitant diseases, physical condition, and prior therapies should be considered before selecting taxane-based treatments for AGC.

## Introduction

Gastric cancer (GC) is one of the leading causes of cancer deaths worldwide, particularly in developing countries^1^. Most patients are diagnosed at advanced stages of GC and usually present with metastasis at diagnosis owing to a lack of health awareness. While radical gastrectomy has shown significant promise as a curative treatment for early GC, it is not satisfactory for cases of advanced GC (AGC)^2^. Therefore, chemotherapy is still a vital treatment for AGC worldwide^3^. For GC patients, particularly those with unresectable local AGC, recurrent GC, or metastatic GC, systemic chemotherapy is the most common treatment option^4, 5^.

Taxanes, a class of drugs including paclitaxel and docetaxel, have been widely used in various systemic chemotherapy regimens for AGC^6, 7^. Paclitaxel (Taxol) is an antileukemic and antitumor agent derived from the bark of the Pacific yew tree *Taxus brevifolia* ^8^. It was initially proven to have an assembly-promoting effect on microtubule proteins which interrupts cell division and proliferation^9–11^. Upon isolation from the needle leaves of the European *Taxus baccata*, docetaxel was characterized by a tricyclic taxane skeleton and antineoplastic activities similar to that of paclitaxel^12^. At present, taxanes are commonly prescribed to treat several cancers and have been shown to have an antitumor effect on lung cancer, GC, breast cancer, and ovarian cancer^13, 14^. Moreover, they have been consistently used in combination with other systemic chemotherapeutic agents including 5-fluorouracil, cisplatin, bevacizumab, and S-1^15–17^.

Some meta-analyses have evaluated the use of paclitaxel and docetaxel against cancers such as advanced non-small-cell lung cancer, prostate cancer, and breast cancer^18–21^. However, to the best of our knowledge, no meta-analysis has evaluated the efficacy and safety of taxanes as palliative chemotherapy for AGC. Since most clinical studies were conducted using either paclitaxel or docetaxel exclusively, a meta-analysis is warranted to evaluate the efficacy and safety of the full taxane drug class for the systemic treatment of AGC.

## Results

### Search results

**Figure 1:**
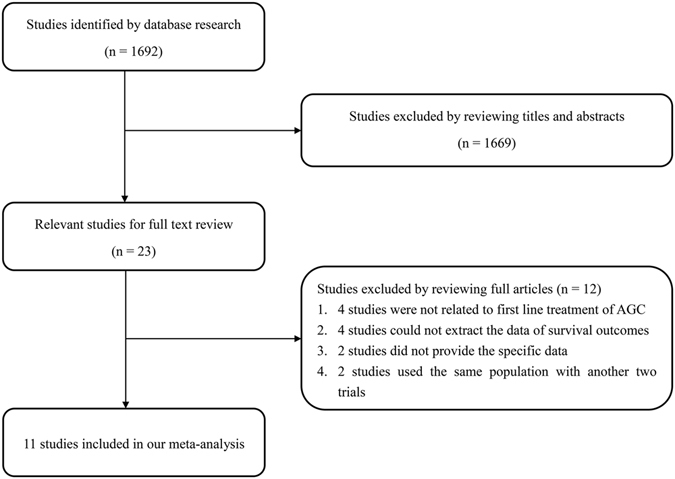
The flow chart of studies selection.

**Figure 2:**
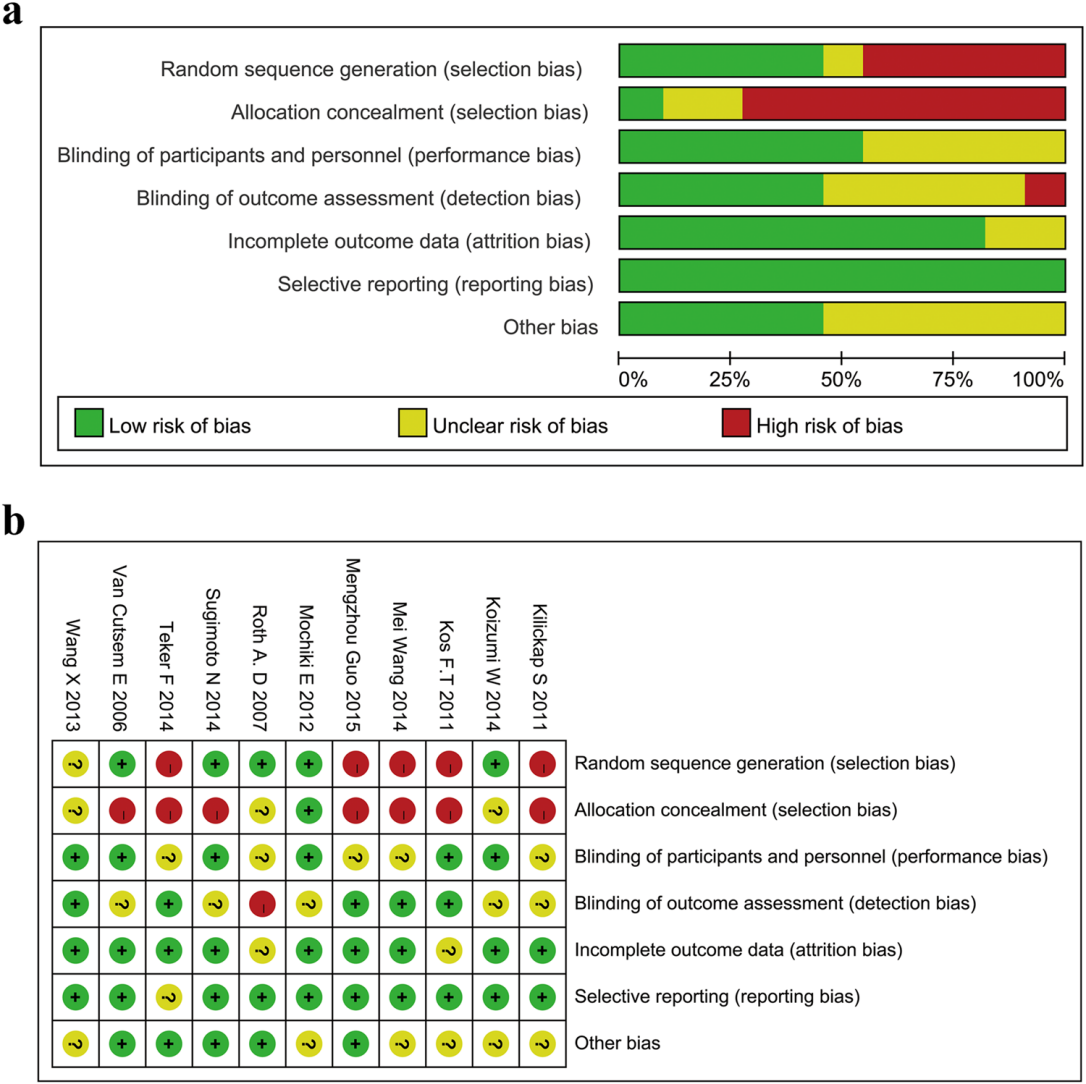
Risk of bias graph and summary of RCTs.

We identified 1692 studies from a database search, and 1654 of these were excluded after reviewing the titles and abstracts. Ultimately, 11 studies met the inclusion criteria (Fig. 1), including 6 randomized controlled trials (RCTs) and 5 non-randomized controlled trials (nRCTs)^22–32^. These trials included a total of 1932 patients, with 969 in the taxane group and 963 in the control group. All patients were diagnosed with AGC, specifically with unresectable local AGC, recurrent GC, or metastatic GC. We obtained data on patients’ history of gastrectomy from four studies and found that approximately 29.4% patients in the taxane group and 27.2% patients in the control group had undergone gastrectomy. All 11 studies investigated the first-line treatment options for AGC. Detailed characteristics are shown in Table 1. The quality of the studies was assessed using the Cochrane risk of bias tool for RCTs and NOS scale for nRCTs, showing that the data was of satisfactory quality to analyze (Fig. 2 and Supplementary Table S1). The quality of the evidence regarding overall survival, progression-free survival, and overall response rate were also evaluated following the GRADE approach and using GRADEpro software (Supplementary Table S2)^33^. Publication bias was observed by performing a funnel plot on ORR (Supplementary Figure S1). Since this test showed signs of bias, we conducted quantitative assessment using Begg’s (p = 0.64) and Egger’s (p = 0.20) tests using Stata software. The trim and fill analysis was also used for testing and adjusting for publication bias in our meta-analysis^34^. We observed that the logRR value (0.166, 95% CI 0.03–0.301, P = 0.017) was similar to the results after trim and fill analysis (0.152, 95% CI 0.018–0.286, P = 0.027), indicating that the results of our study were stable (Supplementary Figure S2). Based on these qualitative results of publication bias, we concluded that the slight publication bias did not affect our overall results.

**Table 1:** Main characteristics of including studies.

Author	Year	Type	Intervention & control		Patient number		Median age (y)		Gender (M/F)		Gastrectory (%)		Outcome measures
			Taxane group	Control group	Taxane group	Control group	Taxane group	Control group	Taxane group	Control group	Taxane group	Control group	
Kilickap	2011	nRCT	DCF	CF	40	36	53/18–70	52/23–70	24/16	18/18	35	40	OS, PFS, ORR, Safety
Koizumi	2014	RCT	DS	S-1	314	321	65/23–79	65/27–79	227/87	229/92	NM	NM	OS, PFS, ORR, Safety
Kos	2011	nRCT	DCF	CF	40	30	52.5/23–68	54.5/35–69	29/11	19/11	NM	NM	OS, PFS, ORR, Safety
Wang	2014	nRCT	DS	SOX	36	48	55.5/33–72	60/35–76	20/16	34/14	NM	NM	OS, PFS, ORR, DCR, Safety
Guo	2015	nRCT	DS	SOX	101	87	60/20–78	56/37–77	59/42	61/26	NM	NM	OS, PFS, ORR, DCR, Safety
Mochiki	2012	RCT	SPac	CiS	42	41	NM	NM	31/11	30/11	21	20	OS, PFS, RR, Safety
Roth	2007	RCT	DCF	ECF	41	40	61/35–78	59/32–71	30/41	30/40	32	18	ORR, Safety
Sugimoto	2014	RCT	SPac	SIri	51	51	62/30–75	64/25–75	28/13	28/13	NM	NM	OS, PFS, ORR, Safety
Teker	2014	nRCT	DCF	ECF	42	44	54/25–72	57/30–77	21/21	28/16	NM	NM	OS, PFS, ORR, Safety
Cutsem	2006	RCT	DCF	CF	221	224	55/26–79	55/25–76	159/62	158/66	NM	NM	TTP, OS, ORR, Safety
Wang	2013	RCT	SPac	S-1	41	41	63/35–74	61/31–73	32/9	30/11	30	32	OS, PFS, ORR, Safety

RCTs: randomized controlled trials, nRCT: non-randomized controlled trials. DCF: Docetaxle, Cisplatin and Fluorouracil; CF: Cisplatin and Fluorouracil; DS: Docetaxel and S-1; SOX: Oxaliplatin and S-1; SPac: Paclitaxel and S-1; CiS: Cisplatin and S-1; ECF: Epirubicin, Cisplatin and Fluorouracil; SIri: Irinotecan and S-1; NM: not mentioned; OS: overall survival; PFS: progression-free survival; ORR: overall response rate; DCR: disease control rate. The quality of RCT were assessed by the Cochrane risk of bias tool, and the quality of nRCT were assessed by Newcastle–Ottawa Scale.

### Overall survival

**Figure 3:**
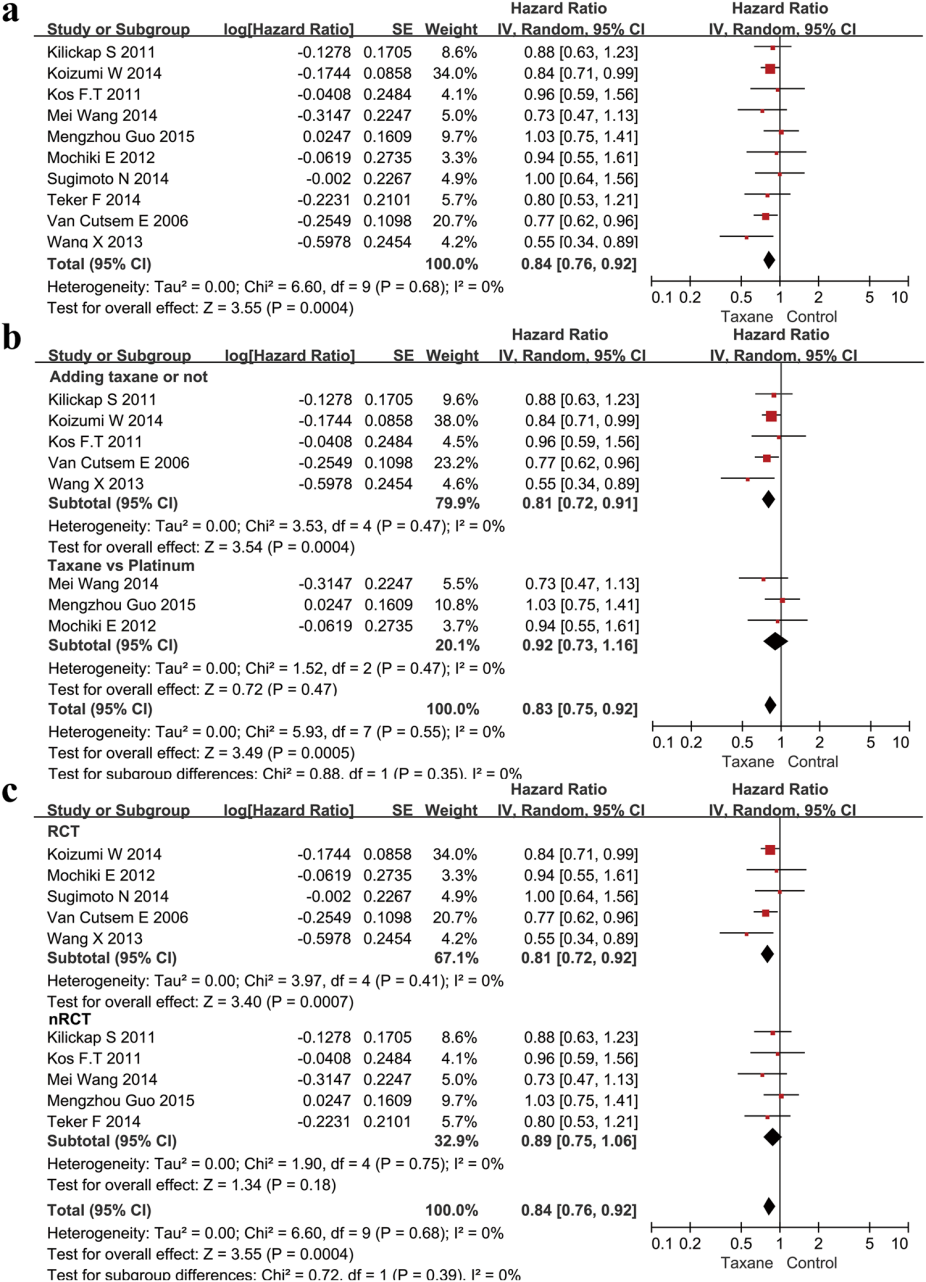
Meta-analysis of the overall survival (OS). (**a**) Forest plots of the hazard ratio (HR) for the OS comparing Taxane with control. (**b**) Subgroup analysis between the adding and replacing groups of the OS. (**c**) Subgroup analysis between the RCT and nRCT groups of the OS.

We extracted OS data from 10 studies (Roth *et al*.^31^ did not analyze OS), including 928 patients in the taxane group and 923 patients in the control group. The results indicated an advantage of taxanes as first-line systemic chemotherapeutic agents for AGC patients compared with other agents (Fig. 3a). The Hazard Ratio (HR) was 0.84 (95% CI 0.76–0.92, P = 0.0004, I^2^ = 0%). We performed a subgroup analysis comparing the different chemotherapy regiments and found that adding a taxane to known chemotherapy regimens had a moderate beneficial effect on OS. The HR was 0.81 (95% CI 0.72–0.91, p = 0.0004, I^2^ = 0%, Fig. 3b). In the comparison between taxane-based chemotherapy and platinum-based chemotherapy, taxane-based chemotherapy trended toward a slight benefit over the platinum-based chemotherapy, but without statistical significance (HR = 0.92, 95% CI 0.73–1.16, p = 0.47, I^2^ = 0%). We also performed a subgroup analysis between the study types. When grouped separately, the RCTs (HR = 0.81, 95% CI 0.72–0.92, p = 0.0007, I^2^ = 0%) and the nRCTs (HR = 0.89, 95% CI 0.75–1.06, p = 0.18, I^2^ = 0%) both showed a benefit when using a taxane, though the result in the nRCT group was not statistically significant (Fig. 3c). We then extracted the median length of the overall survival from the 11 studies and found that 9 of the 11 showed a longer median length of overall survival with a taxane (Supplementary Table S3), further suggesting a benefit of treating with a taxane.

### Progression-free survival

**Figure 4:**
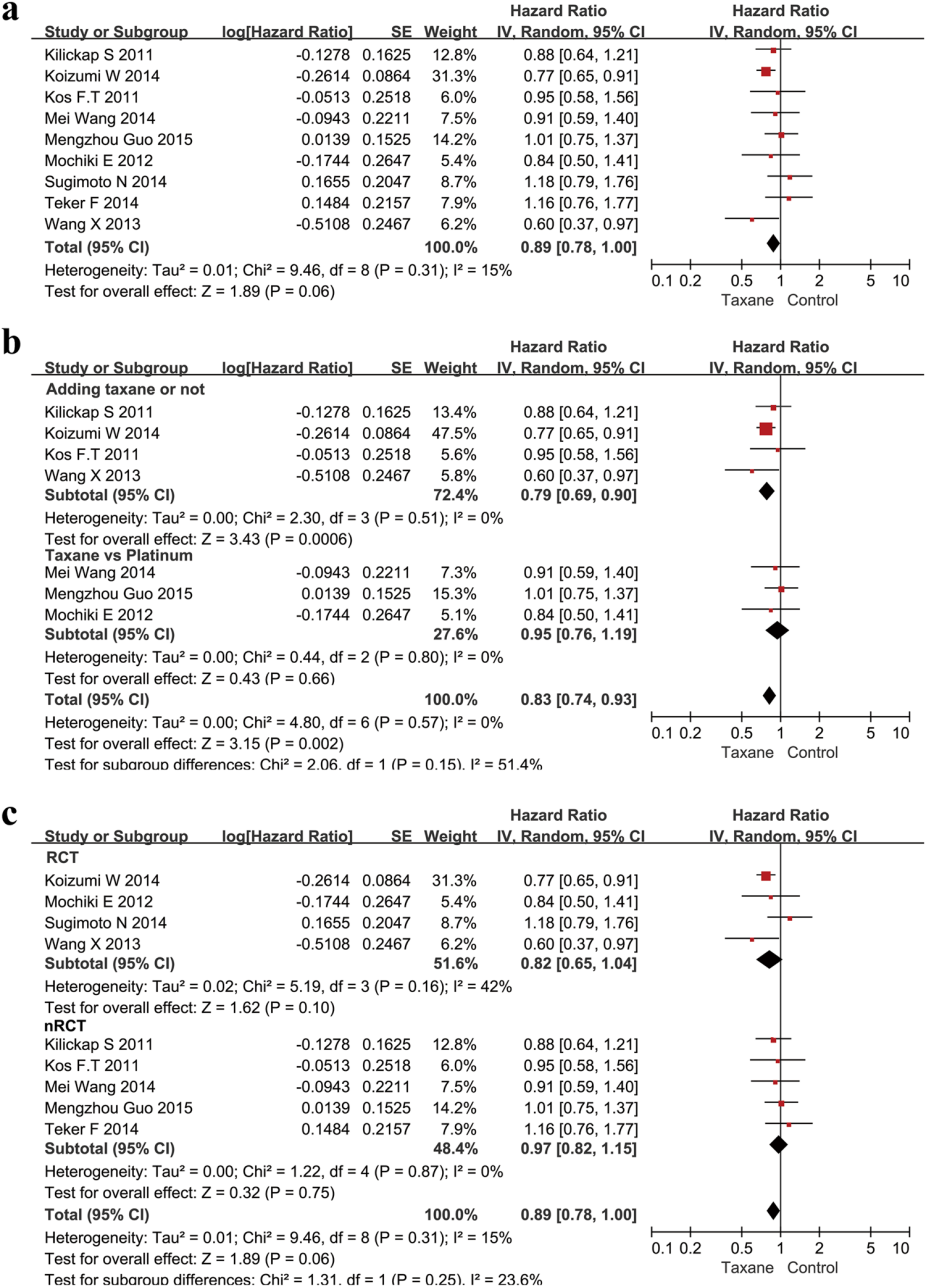
Meta-analysis of the progression-free survival (PFS). (**a**) Forest plots of the hazard ratio (HR) for the PFS comparing Taxane with control. (**b**) Subgroup analysis between the adding and replacing groups of the PFS. (**c**) Subgroup analysis between the RCT and nRCT groups of the PFS.

A total of 1487 patients from nine studies were included in the analysis of PFS. The HR for PFS was 0.89 (95% CI 0.78–1.00, P = 0.06, I^2^ = 15%), indicating that the use of a taxane could prolong the PFS of AGC patients, although the p-value was 0.06 (Fig. 4a). The results of the subgroup analysis indicated that the addition of a taxane improved PFS significantly compared to the original chemotherapy regimens (HR 0.79, 95% CI 0.69–0.90, p = 0.0006, I^2^ = 0%). Taxane-based chemotherapy showed a similar effect compared with platinum-based chemotherapy (HR = 0.95, 95% CI 0.76–1.19, p = 0.66, I^2^ = 0%, Fig. 4b). In the subgroup of RCT publications, taxane-based treatment trended toward improved PFS, but the results did not show statistical significance (HR = 0.82, 95% CI 0.65–1.04, p = 0.10, I^2^ = 42%, Fig. 4c). Longer median progress-free survival was found with taxane use in 7 of 9 studies, indicating the benefit of taxanes for improving the length of progression-free survival (Supplementary Table S2).

### Overall response rate

**Figure 5:**
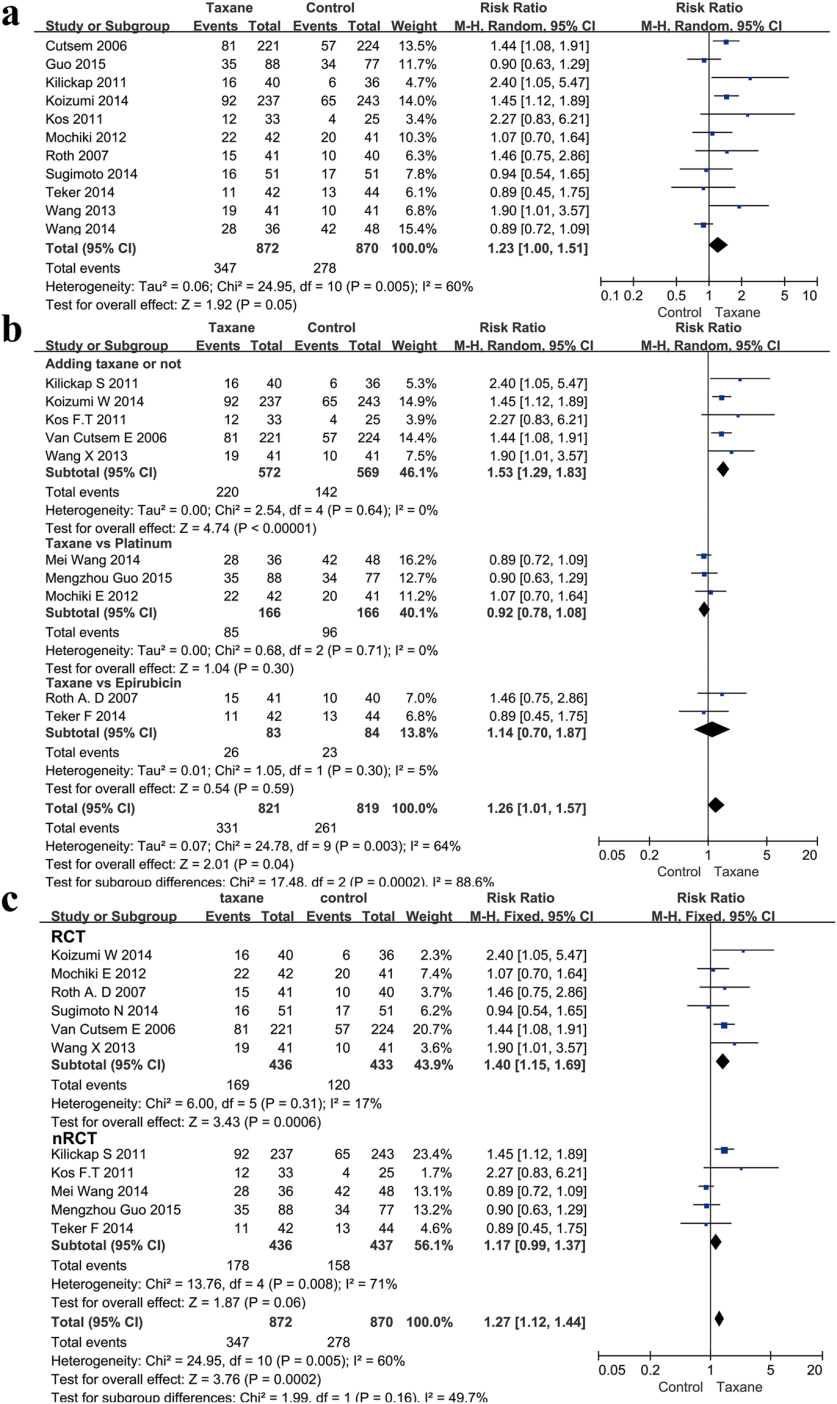
Meta-analysis of the overall response rate (ORR). (**a**) Forest plots of the risk ratio (RR) for the ORR comparing Taxane with control. (**b**) Subgroup analysis between the adding and replacing groups of the ORR. (**c**) Subgroup analysis between the RCT and nRCT groups of the ORR.

All of the included studies contained data on ORR. A total of 1742 patients were included in the analysis: 872 in the taxane group and 870 in the control group. The risk ratio (RR) was 1.23 (95% CI 1.00–1.51, P = 0.05, I^2^ = 60%, Fig. 5a). Our results indicated that using a taxane could lead to a more effective curative effect. The subgroup analysis indicated that simply adding a taxane could significantly improve the ORR (RR 1.53, 95% CI 1.29–1.83, P < 0.00001, I^2^ = 0%, Fig. 5b). We also compared the effect of platinum- or epirubicin-based chemotherapies with taxane-based chemotherapy. The results indicated that taxane–based chemotherapy showed a similar effect both with epirubicin-based chemotherapy (RR 1.14, 95% CI 0.70–1.87, P = 0.59, I^2^ = 5%) and platinum-based chemotherapy (RR 0.92, 95% CI 0.78–1.08, P = 0.30, I^2^ = 0%, Fig. 5b). The results with subgroup analysis both in the RCTs (RR = 1.40, 95% CI 1.15–1.69, P = 0.0006, I^2^ = 17%) and in the nRCTs (RR = 1.17, 95% CI 0.99–1.37, P = 0.06, I^2^ = 71%, Fig. 5c) showed a more effective curative effect by using a taxane.

### Safety

We analyzed the grade 3 and grade 4 toxicities of these studies. The most common hematological toxicities were neutropenia, leucopenia, anemia, and thrombocytopenia. The most common non-hematological toxicities included nausea, vomiting, diarrhea, febrile neutropenia, anorexia, and neuropathy. Taxane-based chemotherapy increased the risk of developing neutropenia and leucopenia when compared to the control group (Table 2). While the risk of developing thrombocytopenia decreased in comparison to control, it was not statistically significant. Compared to platinum-based or epirubicin-based chemotherapy, taxane-based chemotherapy showed no significant advantage or disadvantage in terms of safety. Detailed results are shown in Table 3.

**Table 2:** Toxicities comparison between taxane-based and control chemotherapy.

Toxicities (Grade 3/4)	Study counts	Taxane			Control			RR	95%CI	P value	Model
		Events	Total	Percentage	Events	Total	Percentage				
Neutropenia	11	381	964	39.52%	213	950	22.42%	1.69	1.11–2.56	0.01	Random
Leukopenia	7	241	807	29.86%	102	797	12.80%	2.05	1.05–3.99	0.04	Random
Anemia	9	110	883	12.46%	119	875	13.60%	0.94	0.64–1.38	0.75	Random
Thrombocytopena	9	38	882	4.31%	55	874	6.29%	0.77	0.44–1.35	0.36	Random
Nausea or vomiting	11	142	965	14.72%	135	952	14.18%	1.17	0.80–1.71	0.41	Random
Diarrhea	10	76	923	8.23%	49	908	5.40%	1.45	0.92–2.29	0.11	Random
Febrile neutropenia	6	31	584	5.31%	17	559	3.04%	1.12	0.38–3.28	0.83	Random
Anorexia	7	99	808	12.25%	80	795	10.06%	1.21	0.85–1.72	0.30	Random
Neuropathy & Neurotoxicity	6	33	481	6.86%	21	473	4.44%	1.41	0.49–4.04	0.52	Random

**Table 3:** Toxicities comparison between subgroup.

Toxicities (Grade 3/4)	Adding taxane or not			Taxane-based vs platinum-based				Taxane-based vs epirubicin-based					P value of the interation test
	Study counts	RR	95%CI	P value	Study counts	RR	95%CI	P value	Study counts	RR	95%CI	P value	
Neutropenia	5	2.16	0.89–5.21	0.09	3	1.58	0.90–2.77	0.11	2	1.37	1.03–1.82	0.03	0.06
Leukopenia	3	3.61	1.13–11.51	0.03	2	1.67	0.77–3.61	0.19	NM	NM	NM	NM	0.28
Anemia	4	1.28	0.66–2.48	0.47	3	0.76	0.41–1.40	0.38	NM	NM	NM	NM	0.26
Thrombocytopena	4	0.68	0.36–1.27	0.22	2	0.90	0.15–5.46	0.91	2	0.63	0.08–5.00	0.66	0.95
Nausea or vomiting	5	1.36	0.82–2.27	0.24	3	0.40	0.07–2.31	0.31	2	1.22	0.56–2.63	0.62	0.42
Diarrhea	5	1.35	0.64–2.85	0.43	3	1.58	0.57–4.38	0.38	NM	NM	NM	NM	0.81
Febrile neutropenia	3	1.65	0.18–15.14	0.66	NM	NM	NM	NM	NM	NM	NM	NM	—
Anorexia	3	1.50	0.89–2.52	0.13	2	0.85	0.20–3.60	0.82	NM	NM	NM	NM	0.47
Neuropathy & Neurotoxicity	2	3.23	1.41–7.44	0.006	2	0.40	0.15–1.09	0.07	NM	NM	NM	NM	0.002

NM: Not mentioned.

## Discussion

Various clinical trials have evaluated chemotherapeutic drugs due to their important role in the treatment of AGC. AGC treatment varies slightly in different geographical areas. The National Comprehensive Cancer Network (NCCN) Gastric Cancer Guidelines (Version 2.2016) recommend fluoropyrimidine combined with either cisplatin or oxaliplatin; paclitaxel combined with either cisplatin or carboplatin; docetaxel combined with cisplatin; docetaxel, cisplatin combined with fluorouracil (DCF); or epirubicin and cisplatin combined with fluorouracil (ECF) as first-line chemotherapeutics for unresectable local AGC, recurrent GC, or metastatic GC. The Japanese gastric cancer treatment guidelines (Version 4, 2014) recommend S-1 with cisplatin; S-1 combined with docetaxel; or S-1 and cisplatin combined with docetaxel (DCS) as the first-line treatment^35^. The European Society for Medical Oncology (ESMO) Guidelines recommend epirubicin and oxaliplatin combined with 5-FU (EOF); epirubicin and cisplatin combined with 5-FU (ECF); epirubicin and oxaliplatin combined with capecitabine (EOX); or docetaxel and cisplatin combined with fluorouracil (DCF) as the first-line chemotherapy options for systematic chemotherapy^36^. First-line chemotherapy regimens that include a taxane might be good candidates for the combined treatment of AGC^37^. However, the results of a meta-analysis comparing taxane-based chemotherapy and ECF indicated no benefits of taxanes^38^. Therefore, we aimed to investigate the effect of adding a taxane to known chemotherapy regimens for systemic chemotherapy for AGC.

It has been demonstrated that taxane-based chemotherapy is effective against several types of tumors. In this respect, Tian *et al*. found that the use of a taxane improved short-term local control in Chinese patients with locally advanced nasopharyngeal carcinoma^39^. Moreover, it has been reported that taxanes were beneficial for locally advanced squamous cell carcinomas of the head and neck (SCCHN) and advanced non-small cell lung cancer^40–42^.

We concluded that taxanes significantly improved survival and OS (HR = 0.84, p = 0.0004) compared with the control group in patients with AGC. Moreover, taxanes also have a slight effect on ORR (RR = 1.23, 95% CI 1.00–1.51, p = 0.05), although these effects are not statistically significant. The significant improvement in ORR suggests that patients may be more sensitive to taxane-based treatments. However, the safety-risk analysis uncovered negative effects, indicating that taxanes significantly increased the risk of developing neutropenia and leucopenia.

We performed a subgroup analysis to further the investigation, comparing data from the six RCTs and five nRCTs separately. The RCTs reported that taxanes significantly improved OS, which is similar to the results above. Furthermore, taxanes improved the ORR compared to the control group, suggesting that patients are more responsive to taxane-based treatments than control treatments. The results of the analysis of the RCTs indicated that taxanes increased the risk of developing febrile neutropenia and neuropathy. This result was different from that obtained for the nRCTs, which may be due to the small sample size.

Further subgroup analyses were subsequently performed. One subgroup analysis compared the effect of adding taxanes into known chemotherapy regimens. The results indicated that adding taxanes significantly improved OS, ORR, and PFS, demonstrating that the incorporation of taxanes improved the systemic chemotherapeutic treatment of AGC patients. Another two subgroup analyses evaluated the effects of platinum- or epirubicin-based chemotherapy regimens with taxane-based chemotherapy. Taxane-based chemotherapy did not improve OS, PFS, or ORR when compared with platinum-based chemotherapy, a result which was also observed by Mao *et al*.^43^. Likewise, Roberto P *et al*. found that docetaxel and epirubicin-based chemotherapeutic regimens had similar effects on metastatic gastric cancer^38^. Thus, adding a taxane to known chemotherapy regimens improved the systemic chemotherapeutical treatment of AGC patients, but taxane-based chemotherapy did not have any advantage compared to the platinum- or epirubicin-based chemotherapies.

With regard to toxicity, we found that the inclusion of a taxane increased the risk of neutropenia and leucopenia compared with the original chemotherapy regimens. Alternately, the inclusion of a taxane seemed to decrease the risk of thrombocytopena, but was not statistically significant.

The results of the comparison between single and combined treatment for AGC were similar to those of other studies. Bittoni *et al*. found that first-line triple therapy might be superior to dual therapy for the treatment of AGC patients regarding the ORR and PFS^44^. Mohammad *et al*. also found that first-line triple chemotherapy may be superior to a dual regimen in the treatment of advanced esophagogastric cancer patients^45^. These two studies also found that the triple therapy regimens increased the risk of toxicity, similar to our results. Conversely, Sun *et al*. reported that single-agent treatment should be chosen as the first-line palliative chemotherapy option for older patients with GC^46^. Therefore, the inclusion of a taxane in systemic chemotherapeutic treatments for AGC could improve the therapeutic effect; however, the benefits should be weighed against the risks of treatment-related toxicity.

Similar to the survival analysis results, we did not find significant improvements in safety by replacing the drugs in known chemotherapy regimens with a taxane. However, each drug has unique characteristics. For example, compared with platinum-based chemotherapy, taxane-based chemotherapy increased the risk of neutropenia, leukopenia, and diarrhea but decreased the risk of neuropathy, nausea, and vomiting (Table 3). This result was similar to that of Mao *et al*.^43^. Moreover, taxane-based chemotherapy significantly increased the risk of developing neutropenia but decreased the risk of developing thrombocytopenia compared with epirubicin-based chemotherapy (Table 3). Therefore, replacing chemotherapeutic drugs with a taxane did not significantly decrease the net overall risk of toxicity. For this reason, systemic chemotherapeutic regimens should be chosen according to the patients’ health status and drug characteristics. Further studies should evaluate better combinations of chemotherapeutic drugs. In addition, the effects of newly emerging antineoplastic drugs and their correlation with traditional chemotherapeutic drugs, such as targeted therapies and Chinese medicine^47^, should also be investigated.

Our systematic review and meta-analysis has some limitations. First, the variance in the control group was not uniform due to the limited number of studies that evaluated the use of a taxane alone. Therefore, the heterogeneity might be higher than what was reported herein. Second, some subgroup analyses could not be done due to the limited number of studies, and some of our analyses included only two studies, which might decrease the stringency of the meta-analysis. Third, the quality of the included studies (both RCTs and nRCTs) was poor. Therefore, more high-quality RCTs should be conducted to elucidate the role of taxane in the treatment of AGC.

## Conclusions

The addition of taxane to current first-line treatment options for AGC can improve OS, PFS, and ORR; however, these treatments concomitantly increase the risk of toxicity. The effect of taxane is similar to that of conventional drugs such as oxaliplatin and epirubicin in known chemotherapy regimens. Therefore, other patient characteristics, including concomitant diseases, physical condition, and prior therapies, should be considered before choosing taxane.

## Methods

### Study selection

We searched the PubMed, EMBASE, and the Cochrane Library databases for citations published before February 2016. The keywords searched included “taxane”, “taxol”, “paclitaxel”, “docetaxel”, “gastric cancer”, and “gastric carcinoma”. The full search strategy is shown in the Supplementary Materials. Different search strategies were conducted for different databases, and the references of the included studies were also searched.

### Date extraction and outcomes

The primary outcomes of our study were overall survival (OS), progression-free survival (PFS), and overall response rate (ORR). ORR was defined as the sum of both partial and complete responses. We also included grade 3 and grade 4 adverse events as safety outcomes. Two investigators (Jinxin Shi and Peng Gao) extracted the data from the full articles independently. Any disagreements were resolved by a third investigator.

### Eligibility criteria

Eligibility criteria included (1) studies that were published in English; (2) patients were diagnosed with AGC; (3) studies that evaluated at least one of the three primary outcomes; (4) studies that compared taxane-based therapy with other agent-based therapies as chemotherapy regimens; (5) studies that evaluated first-line chemotherapeutic agents; and (6) in cases of duplicates, the most recent and higher-quality study was included. We excluded case reports, review articles, and letters. The studies were excluded in cases in which none of the outcomes (OS, PFS, ORR, or safety) were provided or could not be calculated, and in cases in which the classification of the chemotherapeutic agents was not provided. Two reviewers (Jinxin Shi and Peng Gao) evaluated the studies independently. The PRISMA 2009 checklist was used as a guideline for reporting the findings for included studies^48^.

### Quality assessment

The quality of the articles were assessed by two researchers independently using the Cochrane risk of bias tool for RCTs and NOS scale for nRCTs^49^. The quality of the evidence used for calculating OS, PFS and ORR was also evaluated using GRADEpro software.

### Statistical analysis

The meta-analysis was performed using Review Manager Software version 5.2 (Cochrane Collaboration). The hazard ratios (HRs) and 95% confidence intervals (95% CIs) of OS and PFS were calculated using the log HR and standard error in Review Manager Software version 5.2 (Cochrane Collaboration) following the method of Tierney^50^. ORR and safety were analyzed by calculating the risk ratio (RR). The random-effects model was selected prior to analysis because it provides more conservative estimates and is tailored to multicenter studies in which heterogeneity is usually present^51^. The p-values less than 0.05 were considered significant. The Begg’s and Egger’s tests were performed using Stata software version 12.0.

## Electronic supplementary material

Supplementary Information
