# Supplementary material for: Efficacy and safety of taxane-based systemic chemotherapy of advanced gastric cancer: A systematic review and meta-analysis
Source: Sci Rep. 2017 Jul 13;7:5319. doi: 10.1038/s41598-017-05464-0 (PMC5509659; doi:10.1038/s41598-017-05464-0)
Supplement: Supplementary file 1 — Supplementary Information [file 41598_2017_5464_MOESM1_ESM.doc]

**Efficacy and safety of taxane-based systemic chemotherapy of advanced gastric cancer: A systematic review and meta-analysis**

Jinxin Shi^1, #^, Peng Gao^1, #^, Yongxi Song^1^, Xiaowan Chen^1^, Yuan Li^1^, Changwang Zhang^1^, Hongchi Wang^1^ and Zhenning Wang^1*^

^1^ Department of Surgical Oncology and General Surgery, First Hospital of China Medical University, 155 North Nanjing Street, Heping District, Shenyang 110001,China

^#^ These authors contributed equally to this work.

*Correspondence to: Zhenning Wang, Department of Surgical Oncology and General Surgery, First Hospital of China Medical University, 155 North Nanjing Street, Heping District, Shenyang 110001, China. Tel: +86-24-8328-3556; Fax: +86-24-2270-3578; E-Mail: [josieon826@sina.cn](mailto:josieon826@sina.cn).

1. Supplementary Information： The actual search strategy is ‘(taxane OR taxol OR paclitaxel OR docetaxel) AND (gastric OR stomach) AND (cancer OR tumor OR carcinoma OR neoplasma)’

2. **Supplementary figure S1 Funnel plot of the studies on the overall response rate (ORR)**


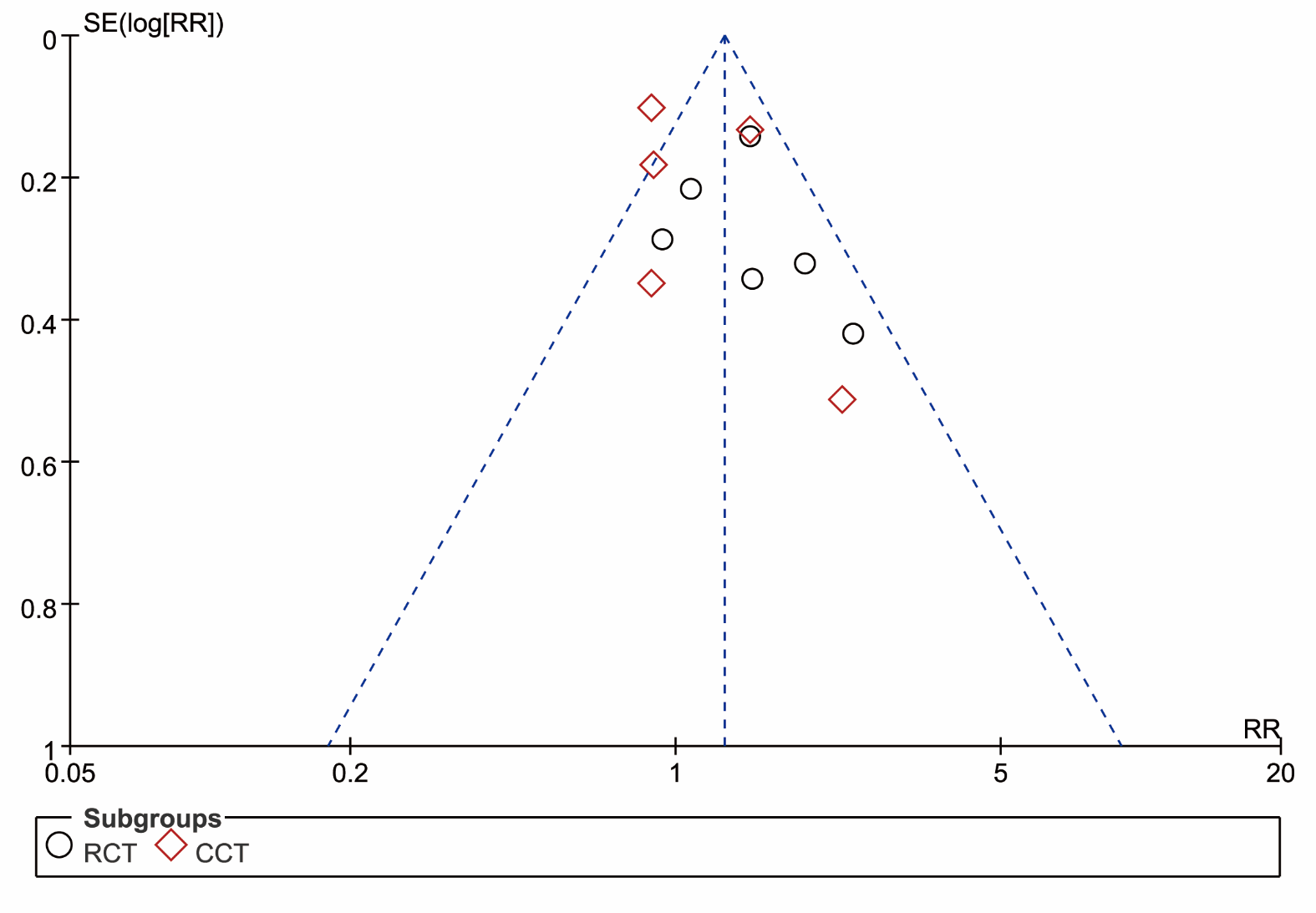


**3. Supplementary figure S2 The results of trim and fill analysis**


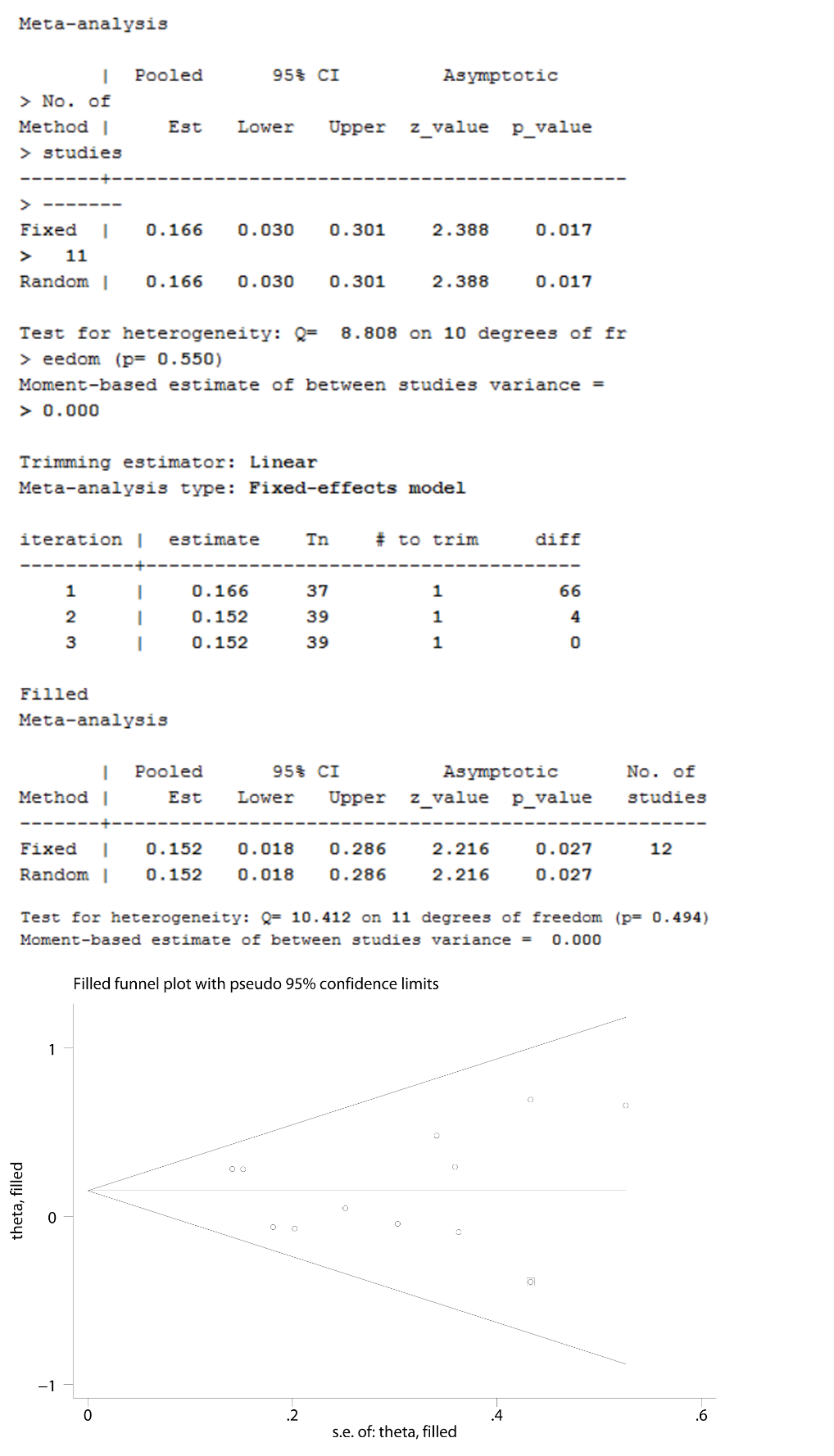


**4. Supplementary table S1 The risk of bias of nRCTS (NOS)**

| **Study** | **Selection** | | | | **Comparability** | | **Outcome** | | | **Total** | **Year** | **Quality** |
| --- | --- | --- | --- | --- | --- | --- | --- | --- | --- | --- | --- | --- |
|  | **REC** | **SNEC** | **AE** | **DO** | **SC** | **AF** | **AO** | **FU** | **AFU** |  |  |  |
| **KilickapS** | **1** | **1** | **0** | **1** | **1** | **0** | **1** | **1** | **0** | **6** | **2011** | **Moderate** |
| **Kos F. T** | **1** | **1** | **0** | **1** | **1** | **0** | **1** | **1** | **0** | **6** | **2011** | **Moderate** |
| **Mei Wang** | **1** | **1** | **0** | **1** | **0** | **0** | **1** | **1** | **0** | **5** | **2014** | **Moderate** |
| **Guo** | **1** | **1** | **0** | **1** | **1** | **1** | **1** | **1** | **0** | **6** | **2015** | **Moderate** |
| **Teker F** | **1** | **1** | **0** | **1** | **1** | **1** | **1** | **1** | **0** | **7** | **2014** | **Moderate** |

Abbreviations: REC: representativeness of the exposed cohort; SNEC: selection of the nonexposed cohort; AE: ascertainment of exposure; DO: demonstration that outcome of interest was not present at start of study; SC: study controls for age, sex; AF: study controls for any additional factors; AO: assessment of outcome; FU: follow-up long enough for outcomes to occur; AFU: adequacy of follow-up of cohorts. “1” means that the study is satisfied the item and “0” means the opposite situation.

| **Quality assessment** | | | | | | | | | **Summary of findings** | | | | | |
| --- | --- | --- | --- | --- | --- | --- | --- | --- | --- | --- | --- | --- | --- | --- |
| **Outcomes（No of studies）** | Limitations | Inconsistency | Indirectness | Imprecision | Publication  bias | Large magnitude  of effect | Dose response | Confounders likely minimize the effect | Number of patients | | Relative risk  (95% CI) | Absolute risk | | Quality |
|  |  |  |  |  |  |  |  |  | Control | Taxane |  | Control risk^a^ | Risk difference  (95% CI) |  |
| **Overall survival** | Serious | No serious inconsistency | No serious indirectness | serious | undetected | no | no | no | 44/854 | 77/852 | OR 1.84 (1.02 to 3.32) | 21 more pre 1000 (1-50) | 40 more pre 1000 (1-102) | ⊕⊝⊝⊝  very low |
| **Progression-free survival** | Serious | No serious inconsistency | No serious inconsistency | No serious inconsistency | undetected | no | no | no | 103/923 | 150/928 | OR 1.51 (1.04 to 2.2) | 55 more pre 1000 (5-119) | 48 more pre 1000 (4-105) | ⊕⊝⊝⊝  very low |
| **Overall response rate** | Serious | No serious inconsistency | No serious inconsistency | No serious inconsistency | undetected | no | no | no | 278/870 | 347/872 | RR 1.23 (1 to 1.51) | 62 more pre 1000 (0-137) | 73 more pre 1000 (0-163) | ⊕⊝⊝⊝  very low |

1. **Supplementary table S2. GRADE evidence proﬁle: Taxane-based chemotherapy for advanced gastric cancer**

Abbreviations: GRADE, Grading of Recommendations Assessment, Development, and Evaluation; RCT, randomized controlled trials; CI, conﬁdence interval; RR, risk ratio.

^a^ The control rate is based on the median control group risk across studies.

1. **Supplementary table S3 Median length of overall survival and progression- free survival**

| Author | OS-Median (95% CI)/month | | PFS-Median (95% CI)/month | |
| --- | --- | --- | --- | --- |
|  | Taxane-based chemotherapy | Chemotherapy without taxane | Taxane-based chemotherapy | Chemotherapy without taxane |
| Kilickap S | 9.6(NM) | 7.4(NM) | 5.8(NM) | 4.3(NM) |
| Koizumi W | 12.5(11.4-14.8) | 10.8(9.5-11.8) | 5.3(4.5-5.9) | 4.2(3.7-4.7) |
| Kos F. T | 8.7(6.7-10.7) | 6.5(1.8-11.2) | 6.2(5.6-6.8) | 4.4(1.8-7.0) |
| Mei Wang | 13.97 (NM) | 13.13 (NM) | 6.55(NM) | 5.73(NM) |
| Mengzhou Guo | 13.1(NM) | 12.8(NM) | 5.8(NM) | 5.5(NM) |
| Mochiki E | 16(15-22) | 17(11-23) | 9(6-12) | 6(4-9) |
| Roth A. D | 10.4（8.3-12.0） | 8.3（7.2-13.0） | NM | NM |
| Sugimoto N | 12(10.2-18) | 12.5(9.2-16.7) | 4.6(4.1-6.0) | 5.7(4.4-8.1) |
| Teker F | 11.0(9.1-12.8) | 10.0(8.2-11.7) | 6.0(5.3-6.6) | 6.0(4.9-7.0) |
| Van Cutsem E | 9.2(8.4-10.6) | 8.6(7.2-9.5) | NM | NM |
| Wang X | 14.0(7.2-20.8) | 11(5.1-18.6) | 6.0(3.8-11.6) | 4.0(2.7-8.9) |

NM，not mentioned
